# Edinburgh Royal Infirmary

**Published:** 1894-03-17

**Authors:** 


### EDINBURGH ROYAL INFIRMARY.

### Catarrhal Jaundice and its Treatment.

The term jaundice, like its medical synonym icterus, refers solely to the yellow colouration of the skin which forms such a marked feature of this affection, which does not therefore rank as a disease *per se*, but forms a symptom of a number of diseases greatly differing in severity. In speaking of catarrhal jaundice we refer to the condition known to nosologists as *icterus simplex*, which from its frequency has come to be popularly known by the name of its chief symptom.

It is an affection of which our knowledge is derived almost solely from a clinical standpoint, as it is not a fatal disease, and death during its course from other causes is rare. It is due to the absorption of the bile into the blood, owing to some temporary obstruction to its outflow from the bile-ducts, this being believed to result from a catarrhal condition of the duodenum itself, or of the larger ducts, and it is uncertain whether or not a complete closure of the ducts is necessary. It is at least possible that a comparatively slight resistance may be sufficient, as bile is secreted at low pressure. The pigment is carried by the blood stream and deposited in the skin and mucous membranes, and is excreted by the kidney, giving the urine a characteristic olive-green colour. The bile acts powerfully on the nervous system (probably by its acids), and produces a remarkable slowing of the pulse rate, which often falls to 40 per minute. There is also in many cases a very troublesome itching of the skin, and it is not uncommon to observe a slight elevation of temperature for a few days. At the same time the bile is not poured into the intestinal canal, so that the motions become clay-coloured from the absence of bile pigment and the presence of imperfectly assimilated fats. They are also very offensive from the decomposition normally kept in check by the antiseptic properties of the bile.

It is evident that such an attack of catarrhal jaundice is a possible complication of a gastro-duodenal catarrh, such as frequently results from the ingestion of irritating food, alcoholic excess, tobacco, or exposure to cold. The treatment must, therefore, be directed to the improvement of the duodenal condition, this being usually done by the administration of such drugs as bismuth and rhubarb along with the sulphate or bicarbonate of soda. The diet must be regulated by the exclusion of fatty substances, and should be otherwise liberal, though plain. The state of the bowels should also receive careful attention, and for this purpose salines are to be preferred. Confinement to bed is not necessary, as exercise is beneficial.

The itching of the skin may be relieved, if very severe, by sponging with warm alkaline lotions, as a solution of bicarbonate of soda.

After a fortnight or three weeks the intestinal evacuations resume their natural colour, and this is followed by the disappearance of the pigment in the urine, but its removal from the skin is much more gradual.

Somewhat akin to the catarrhal form, two other "benign" varieties of jaundice may be mentioned—that from mental emotion and that from suppression of a chronic hæmorrhagic discharge.

Cases of the first variety have been frequently recorded where a person, after violent emotion, such as grief or anger, has become rapidly jaundiced.

The pathology of this state is obscure, and it has been suggested that the bile may be forced into the venous radicles of the liver. The other form is that where on the sudden arrest of the bleeding from hæmorrhoids or the suspension of menstruation by chills or other causes, jaundice has rapidly supervened, the mechanism being conjectured to be a sudden engorgement of the liver with blood.

Both these varieties are usually of short duration, and tend to spontaneous recovery, and may, therefore, be fitly classed with simple jaundice as distinguished from those conditions where the pigmentation is but a symptom of a grave, progressive, and frequently fatal affection.

